# Self-Management Behavior in Patients with Type 2 Diabetes: A Cross-Sectional Survey in Western Urban China

**DOI:** 10.1371/journal.pone.0095138

**Published:** 2014-04-17

**Authors:** Mingjun Huang, Rui Zhao, Sheyu Li, Xiaolian Jiang

**Affiliations:** 1 Department of Gastrointestinal Surgery, West China Hospital, Sichuan University, Chengdu, Sichuan, China; 2 Nursing Faculty of West China, Sichuan University, Chengdu, Sichuan China; 3 Endocrinology Department, West China Hospital, Sichuan University, Chengdu, Sichuan, China; 4 Nursing Department, West China Hospital, Sichuan University, Chengdu, Sichuan, China; University of Tolima, Colombia

## Abstract

**Purpose:**

To investigate the current status of diabetic self-management behavior and the factors influencing this behavior in Chengdu, a typical city in western China.

**Methods:**

We performed stratified sampling in 6 urban districts of Chengdu. We used questionnaires concerning self-management knowledge, self-management beliefs, self-management efficacy, social support, and self-management behavior to investigate patients with T2DM from August to November 2011. All of the data were analyzed using the SPSS 17.0 statistical package.

**Results:**

We enrolled a total of 364 patients in the present study. The median score of self-management behavior was 111.00, the interquartile range was 100.00–119.00, and the index score was 77.77. Self-management was described as “good” in 46%, “fair” in 45%, and “poor” in 6% of patients. A multiple-factor analysis identified age (OR, 0.43; 95% CI, 0.20–0.91; *P* = 0.026), education in “foot care” (OR, 0.42; 95% CI, 0.18–0.99; *P* = 0.048), self-management knowledge (OR, 0.86; 95% CI, 0.80–0.92; *P*<0.001), self-management belief (OR, 0.92; 95% CI, 0.87–0.97; *P* = 0.002), self-efficacy (OR, 0.93; 95% CI, 0.90–0.96; *P*<0.001), and social support (OR, 0.62; 95% CI, 0.41–0.94; *P* = 0.023) as positive factors. Negative factors included diabetes duration (5–9 years: OR, 14.82; 95% CI, 1.64–133.73; *P* = 0.016; and ≥10 years: OR, 10.28; 95% CI, 1.06–99.79; *P* = 0.045) and hospitalization experience (OR, 2.96; 95% CI, 1.64–5.36; *P*<0.001).

**Conclusion:**

We observed good self-management behavior in patients with T2DM in Chengdu. When self-management education is provided, age, education, knowledge, belief, self-efficacy, and social support should be considered to offer more appropriate intervention and to improve patients' behavior.

## Introduction

In mainland China, the population of Type 2 diabetes mellitus (T2DM) is predicted to reach 42.3 million in 2030[1]. Notably, 78% of diabetic patients in China suffer from more than one complication [2]. T2DM and its complications pose an enormous threat to our health status and represent a heavy economic burden for individuals and for society in general [3].

To lower the morbidity of diabetes complications, self-management is critical [4]–[5]. Self-management, proposed by Barlow, refers to therapeutic alliance, monitoring disease signs and symptoms, maintaining and improving health behavior, and addressing the negative impact of the disease on the patients' physical function, emotional feelings, and interpersonal relationships [6]. A Finnish study suggested that good self-management could improve or control blood glucose values and reduce the severity of complications [7]. Another study reported that in a Veterans Affairs Hospital, the reduced amputation rate attributable to self-management education translated to a cost savings of $378,000 over 2 years [8].

Generally, self-management education has been acknowledged as the key to helping patients with T2DM achieve better self-management [7], [10]. However, despite the encouraging reports, the status of self-management education is insufficient in western urban populations in China [4]. However, the effectiveness of specific diabetes education for specific populations has not been identified [11]. Few systematic studies have evaluated the influencing factors in T2DM in western China, although such factors have been evaluated and tested in the US [12]. In western China, few resources for diabetic education are available compared with those available in the US and Japan. The community nursing/residents ratio is 1∶16745 throughout China as a whole, whereas in western China, the ratio is 1∶26384 [13]. Both of these values are lower than the WHO standard ratio of 1∶12600 [13]. Additionally, physicians and nurses, who are primarily responsible for providing diabetes self-management education in China, are less well trained than in western nations, and the diabetic self-management education they provide is haphazard [14]. In the US, self-management is often recommended in accordance with the national standards and is provided by professional diabetes educators [15].

The present study aimed to investigate the current status of diabetic self-management in Chengdu, a typical city in western China.

## Materials and Methods

### Study design

#### Stratified sampling

One community was adopted in every district (total  = 6) of Chengdu: the Yulin community in Wuhou District, the Supo community in Qingyang District, the Longzhoulu community in Jinjiang District, the Caojiaxiang community in Jinniu District, the Shuanglin community in Chenghua District, and the Guixi community in Gaoxin District. The samples were extracted randomly using a computer system in proportion to the population in each community (community population, extraction object  = 1000∶1). The eligible research patients were all informed of the study procedures, and informed consent was obtained before the patients completed the questionnaires. The patients' personal data were only used in the research and were secured as research data. The study was approved by the *Ethics Committees for Clinical Trials and Biomedical Reaearch in West China Hospital, Sichuan University*, and the study number is 2013–159.

### Patients

From August to November 2010, patients with T2DM who lived in the urban communities of Chengdu, China, were over 18 years, and had been diagnosed more than one month prior according to the 1999 World Health Organization (WHO) diagnostic criteria were enrolled in the study [16]. The participants were expected to live in the investigated community in the ensuing 6 months. Patients with severe cognitive disorders or mental disorders were not included in this study. Additionally, patients were excluded if they were pregnant, suffered from cancer or organ failure, or refused to participate in the research study. All of the investigations were performed by well-trained nurses.

### Data collection

The collecting sites were primarily located in community health centers; minimal data were collected in the patients' households. The questionnaires adopted the unified guidance method and were completed by the patients. For those who could not complete the form themselves, the investigators recorded and verified the patients' options after reading the questions and choices to them. All of the questionnaires were retrieved immediately after completion. The questionnaires in the study are described below.

T2DM patients' self-management knowledge was accessed using a questionnaire, adapted by the research team, that was based KAP (Knowledge-Attitude-Practice) theory [15], [17]. The questionnaire included 46 items and 4 dimensions: knowledge of risk factors, symptoms, complications, and health management. With respect to the scoring method, “Yes” responses were recorded as 1 point, “No” or “Uncertain” responses were recorded as 0 points, and negative items used reverse scoring. Higher scores represent more knowledge. The content validity ratio (CVR) of the scale was 0.965. For each dimension, the CVR was 0.900–1.

Self-management beliefs were measured using a diabetes-specific scale. The scale, developed on the basis of the HBM (Health Belief Model) and created by Chen in China [18], comprises 5 subscales and 20 items and uses a 5-point Likert scale; higher scores represent better beliefs. The content validity was 0.81, the test-retest reliability was 0.78–0.82, and Cronbach's α was 0.79.

A six-item chronic disease self-management efficacy scale, derived from the “Chronic Disease Self-Management Program Questionnaire Code Book” published by the Stanford Patient Education Center in 2007 [19], assessed the patients' self-management efficacy using a linear scale from 1 to 10. Higher scores indicate better efficacy. The internal consistency reliability of the scale was 0.9.

Social support, measured by the well-validated social support rating scale (SSRS) created by Xiao [20], comprises 10 items and 3 subscales: objective support, subjective support, and degree of social support utility. Higher scores indicate better support. The test-retest reliability coefficient and the consistency of scale were 0.92 and 0.88–0.94, respectively.

The scale used to access the patients' self-management behavior was adapted from the scale developed by Hurley and Shea and revised by Wang[21]. This scale comprises 28 items and 6 subscales: drug management, blood sugar monitoring, foot care, sports management, prevention and treatment of hyperglycemia/hypoglycemia, and total behavior management. The scale employs a 5-point Likert scale. Higher scores indicate better behavior. The construct validity of the original scale was 0.68, and Cronbach's α was 0.87. The CVR of the scale was 0.93; for each dimension, the CVR ranged from 0.73 to 1. The reliability coefficients of the internal consistency (Cronbach's α) of the five scales in the pre-survey were 0.88, 0.82, 0.89, 0.70 and 0.89, respectively.

### Statistical analysis

According to the requirements of multivariate analysis and after consulting statisticians (sample size as the independent variable of 10 to 20 trials), 16 independent variables and 320 patients (16×20 = 320) were calculated to be necessary for the research. In light of sample loss, a sample number of 384 was selected, representing an increase of approximately 20%. SPSS 17.0 (SPSS Inc., Chicago, IL, USA) was used to establish the database and perform the analysis. The mean ± standard deviation (M± SD), median, interquartile range, frequency, and composition ratio were used to describe the statistical results. With respect to statistical inference, nonparametric tests (Mann-Whitney U test and Kruskal-Wallis H test), Spearman rank correlation, and two categories of non-criteria logistics regression analysis were used. The study evaluated the results using a conventional statistical criterion (α = 0.05) and two-sided Lan-DeMets monitoring boundaries.

## Results

### Baseline characteristics of the patients

Of a total of 384 patients, 376 questionnaires were retrieved, including 364 questionnaires that were valid (the response rate of valid questionnaires  = 95%). The majority of the participants were females, and the mean age was 67.87±9.50 years. The majority of the participants were of Han ethnicity, and 77% were limited to below junior college-level education.

### Score of T2DM self-management behavior

The median behavior score was 111.00, and the score index was 78%. The lowest behavior score item was “prevention and treatment of hyperglycemia and hypoglycemia” (68%), whereas the “drug management” behavior score was the highest (91%). The behavior of 46% patients was described as “good”, and 45% were described as “fair” (Tables 1 and 2).

**Table 1:** Scores of T2DM self-management behavior for each dimension (N = 364).

Items	Score Interval	Median	Quartile Range	Index Score (%)???
Drug Management	3–15	15.00	13.00–15.00	91.39
Diet Management	6–30	25.00	23.00–28.00	81.68
Blood Sugar Monitoring	4–20	17.00	14.00–18.00	79.88
Foot Care	5–25	20.00	18.00–22.00	77.37
Exercise Management	4–20	16.00	12.00–19.00	74.99
Prevention and Treatment of Hyperglycemia/Hypoglycemia	6–30	20.00	17.00–24.00	67.71
Total Behavior Management	28–140	111.00	100.00–119.00	77.77

? The index scoring  =  (actual total score/possible highest score) ×100%.

**Table 2:** Grade of T2DM self-management behavior for each dimension (N = 364)△.

Items	Poor (n, %)	Fair (n, %)	Good (n, %)
Drug Management	22 (6.04)	104 (28.57)	238 (65.38)
Diet Management	70 (19.23)	93 (25.55)	201 (55.22)
Blood Sugar Monitoring	10 (2.75)	27 (7.42)	316 (86.81)
Foot Care	44 (12.09)	80 (21.98)	240 (65.93)
Exercise Management	27 (7.42)	152 (41.76)	185 (50.82)
Prevention and Treatment of Hyperglycemia/Hypoglycemia	104 (28.57)	162 (44.51)	98 (26.92)
Total Behavior Management	22 (6.04)	163 (44.78)	168 (46.15)

△ Score index: scoring <60% represents “poor”; 60%–80% indicates “fair”; and ≥80% implies “good”.

### Single factor analysis of T2DM self-management behavior

#### Relationship between patient characteristics and T2DM self-management behavior

The nonparametric test (due to skewed distribution data, the results were described and illustrated by the median of the behavior score) revealed that there were significant differences (*P*<0.05) among the patients' self-management behavior scores according to the patients' age, education, occupation, income, method of paying medical expenses, duration of diabetes, and number of times they received health education (Table 3).

**Table 3:** Relationship between patient characteristics and self-management behavior.

	Group	N	Constitution Ratio (%)	Median	Z/H	*P*
Gender	Male	156	42.86	109.00	−0.32???	0.751
	Female	208	57.14	112.00		
Age (year)	<60	64	17.58	104.00	9.24^△^	0.010
	60–79	265	72.80	110.00		
	≥80	35	9.62	113.00		
Marriage	Unmarried	7	1.92	103.00	0.66^△^	0.720
	Married/remarried	282	77.47	111.00		
	Divorced/widowed	75	20.60	111.00		
Education	Primary and below	94	25.82	107.00	8.04^△^	0.018
	Middle school	188	51.64	113.00		
	Junior college and above	82	22.52	108.00		
Occupation	Retired	299	82.14	112.00	25.80^△^	<0.001
	Employed	47	12.91	104.00		
	Unemployed	18	4.95	93.00		
Income(yuan/month/person)	<1000	73	20.06	100.00	26.67^△^	<0.001
	1001–2000	211	57.96	112.00		
	>2000	80	21.98	114.00		
Method of paying medical expenses	Public expense	13	3.57	100.00	8.01^△^	0.018
	Social/commercial insurance	336	92.31	111.00		
	private expense	15	4.12	99.00		
Duration of diabetes (years)	1–4	1160	43.96	114.00	24.67^△^	<0.001
	5–9	336	24.45	112.00		
	≥10	115	31.59	107.00		
Hospitalization experience	No	214	58.79	112.00	−1.39???	0.165
	Yes	150	41.21	109.00		
Complications	No	172	47.25	111.00	−0.14???	0.886
	Yes	192	52.75	111.00		
Treatment	Diet	11	3.02	108.00	1.09^△^	0.297
Modality	Diet + Insulin/antidiabetic	282	77.48	110.00		
	Diet + insulin + antidiabetic	71	19.50	113.00		
Number of instances of health education	0	122	33.52	105.00	40.83^△^	<0.001
	1–4	127	34.89	110.00		
	≥5	115	31.59	118.00		

? Mann-Whitney U test; ^△^Kruskal-Wallis H test.

#### Correlation analysis among self-management behavior, self-management knowledge, self-management belief, self-efficacy, and social support

The correlation analysis revealed that there were positive correlations between self-management behavior (total and subscale scores) and knowledge, belief, self-efficacy, and social support (Table 4).

**Table 4:** Correlation analysis between behavior and knowledge, belief,self-efficacy and social support (N = 364).

	Total score	Diet management	Sports management	Drug management	Blood glucose monitoring	Foot care	Prevention and Treatment of Hyperglycemia/Hypoglycemia
Knowledge of self-management	0.47**	0.23**	0.24**	0.21**	0.35**	0.34**	0.42**
Belief of self-management	0.47**	0.30**	0. 20**	0.33**	0.38**	0.37**	0.31**
Self-efficacy	0.38**	0.23**	0.33**	0.24**	0.22**	0.30**	0.19**
Social support	0.20**	0.06	0.23**	0.01	0.10*	0.09	0.21**

The data are non-normally distributed; therefore, Spearman rank correlation was used. * *P*<0.05; ** *P*<0.01.

### Multiple-factor analysis of T2DM self-management behavior

Self-management behavior was divided into two groups using the score index: <80% indicates “not good” and >80% indicates “good” [18]. The variables evaluated are shown in Table S1. The multiple-factor analysis indicated that the duration of diabetes and hospitalization experiences were negative impact factors for self-management behavior, whereas age, education, self-management knowledge, self-management belief, and social support exerted a positive influence on self-management behavior (Table 5).

**Table 5:** Multiple-factor analysis of self-management behavior.

Impact factors	B	S.E.	*P*	OR (95% CI)
Age	−0.85	0.38	0.026	0.43 (0.20–0.91)
Education	−0.87	0.44	0.048	0.42 (0.18–0.99)
Diabetes duration (5–9 years)	2.70	1.12	0.016	14.82 (1.64–133.73)
Diabetes duration (≥10 years)	2.33	1.16	0.045	10.28 (1.06–99.79)
Hospitalization experience	1.09	0.30	<0.001	2.96 (1.64–5.36)
Knowledge	−0.16	0.04	<0.001	0.86(0.80–0.92)
Belief	−0.08	0.03	0.002	0.92 (0.87–0.97)
Self-efficacy	−0.07	0.02	<0.001	0.93 (0.90–0.96)
Social support	−0.48	0.21	0.023	0.62 (0.41–0.94)

## Discussion

The study revealed that the score index of the patients' self-management behavior and the number of patients whose self-management was graded as “good” are both higher than the figures reported by several previous articles in China [22]–[23]. Multivariate factor analysis suggested that the influence that the patients' general data and the duration of diabetes exerted on self-management was consistent with the practical value of the knowledge, belief, self-efficacy, and self-management behavior, as the KAP and HBM theories had indicated. This study will help in the promotion of the self-management of patients with T2DM by providing information regarding the effects of health education in Chengdu, western China.

The patients in the present study exhibited good self-management behavior. This result is partially attributed to the quick development of the primary healthcare system and the community health centers in urban areas of China [24]. The healthcare system has been established gradually and has highlighted the management of chronic diseases, including diabetes [25]. However, the patients in our study exhibited uneven performances in various dimensions of self-management behavior. Better self-management was observed in the administration of medication and diet management, whereas the prevention and management of hyperglycemia/hypoglycemia were poor. These findings, consistent with several prior research studies [16], [26], illustrate the differences that exist in the persistence and performance of self-management behavior among patients with T2DM. Thus, T2DM educational providers should aim to address the weaknesses of patients' behavior after assessment after considering the characteristics of the population of the province in question, instead of blindly adhering the findings of other districts.

Multivariate factor analysis indicated that elderly patients or those with a higher-level education exhibit better self-management behavior. Young patients, who are engaged in their careers, are busy engaging in social interactions; these patients tend to have more active social lives and to spend less time and energy managing their disease regularly [27]. Therefore, for young patients, it is essential to enhance self-management consciousness. A higher level of education also exerts a significant influence. A national study in China indicated that patients with a higher education have a more positive attitude towards their diabetes and tend to achieve better blood glucose control [16]. Highly educated people are more capable of receiving and handling knowledge [16]. The KAP model mentions that “knowledge” is the basis of behavior [28] and the lack of knowledge or misunderstanding may be the primary reasons for unhealthy behavior [19]. Meanwhile, the patients with a lower educational background, according to the investigation, were more likely to have the misconception that hypoglycemia was not a concern for diabetic patients, which hinders the adoption and implementation of preventing and managing behavior. When health education is provided to patients with lower educational levels, imparting health knowledge and skills should be highlighted.

We observed that the long-term duration of diabetes and hospitalization experiences also negatively impacted self-management behavior. Maintenance of stable blood glucose relies on the long-term use of medications [9], [29]. Because the duration of diabetes may be long, patients can become frustrated from the long-term use of medications and continuous self-management and might gradually ignore self-care [9]. Several patients may change their medication behavior due to callosity in the injection site, hypersusceptibility, or a decrease of drug efficacy [30]. Furthermore, physical well-being, mentioned in Horsburgh's study [31], was fundamental to performing self-care; the attitude towards self-care will worsen with the severity of the disease and complications. Patient attitude, according to KAP theory, is instrumental in changing behavior; self-management ability will be weakened if the patients holds a laissez-faire attitude [32]–[34]. Additionally, the patients who experience repeated hospitalizations, generally with severe complications and poorly controlled blood glucose, will feel more management obstacles attributable to their poor health. More importantly, poor self-management behavior likely leads to the increasing need for hospitalization, which indicates that diabetes education during patients' hospitalization in western China is presently in its initial developmental stages. Although patients with long-term duration of diabetes tend to understand more about their disease, healthcare providers should focus on assessing and strengthening the self-management education of patients with T2DM.

We observed that self-management will be promoted by enriched self-management knowledge, positive self-management beliefs, and high self-efficacy, which was consistent with the findings of other researchers. Corbett [35] and Chang [9] noted that diabetes-related knowledge, which is fundamental to the effective self-management of diabetic patients, directly influences patient compliance and enables patients to self-manage their behavior. A cross-sectional survey by Whittemore noted that a higher score for health beliefs translates into better self-management behavior in diabetic patients [36]. A study led by Benjamin on elderly patients who suffered from multiple chronic diseases indicated that medicine-efficacy believers adhere to medications more closely than those who hold a negative view [37]. McDonald-Miszczak reported that a negative belief will function as a passive guiding role in self-management behavior [38]. KAP theory indicates that knowledge is basic to building a positive belief, maintaining good behavior, and changing harmful habits [15]. According to HBM, people will persevere in a healthy lifestyle or forsake bad habits once they perceive their susceptibility to the disease, the severity of the disease, the disease risk factors, or the benefits and obstacles of changing.

Self-efficacy associated with individual capacity, a decisive factor of human behavior, refers to the capacity of judgment and an individual's beliefs or subjective understanding and feelings towards his or her actions. Patients with T2DM must form long-term habits of diet, exercise, foot care, medication administration, insulin injection, and blood glucose monitoring [39]. This transformation of behavior demands the capabilities, confidence, and determination of patients, based on their self-assessment, which is likely the reason why high self-efficacy results in a better perseverance of self-management [40]–[41]. The results validate KAP theory and the HBM, as well as the positive effect of self-efficacy, and these results remind us that when disseminating diabetes education, the educators should not only explain and share knowledge, as the basis of behavioral changes, but should also cultivate the correct beliefs and attitudes, in addition to boosting patients' self-efficacy, to promote the transformation and implementation of effective self-management.

Social support boosts self-management behavior. King's study revealed that better social support will result in a better perseverance of self-management [42]. Social support is the material and spiritual support that an individual gains from his or her own social relationships to reduce psychological stress, relieve tension, and improve social adaptability [43]. Higher levels of social support facilitate self-efficacy [44] because self-efficacy contributes to an individual's capabilities. These findings have been confirmed in several studies [40], [45], [46]. Research by Schiøtz verified that there is a positive correlation between the social support of patients with T2DM and self-efficacy [47]. Thus, if material assistance, emotional support, and spiritual encouragement are available, the patients will be more determined to support self-management and to fight the disease. To promote T2DM patients' self-management, health promoters should encourage not only the patients' self-efficacy but also their confidence and, more importantly, strengthen their social support.

### Limitations

Several limitations of the study should be noted. First, because this is a cross-sectional study, it is difficult to prove causal relationships among the factors. Second, there are selection biases in our study, such as nonresponse bias (response rate, 94.79%). Although Chengdu is a typical city in western China, another city was not included in the research; therefore, the potential selection bias cannot be avoided. Although there are many influential factors listed, other variables, such as depression, which is associated with the self-management of patients with T2DM, have not been fully recognized. Additional studies should be conducted with a larger sample size and improved research methodologies.

Of all the factors influencing the self-management behavior of T2DM in Chengdu, age, education, self-management knowledge, belief, self-efficacy, and social support were identified as positive factors. When providing information on the self-management of T2DM, age, education, knowledge, belief, self-efficacy, and social support should be evaluated to offer appropriate intervention and improve patient behavior. Additional research is warranted to assess more influencing factors of patients with T2DM on a larger scale and to eliminate the interference of the psychological status of diabetes patients. Future research should focus on determining the causality between the influencing factors and self-management behavior.

## Supporting Information

Table S1 The variables and the evaluation of logistic regression analysis is in the Supplementary data.
(DOCX)
