# Supplementary material for: Self-Management Behavior in Patients with Type 2 Diabetes: A Cross-Sectional Survey in Western Urban China
Source: PLoS One. 2014 Apr 17;9(4):e95138. doi: 10.1371/journal.pone.0095138 (PMC3990599; doi:10.1371/journal.pone.0095138)
Supplement: Table S1 — The variables and the evaluation of logistic regression analysis is in the Supplementary data. (DOCX) [file pone.0095138.s001.docx]

**Supplementary data:**

**Table S1 Variables and the evaluation of logistic regression analysis**

|  | Variable name | Evaluation |
| --- | --- | --- |
|  | **Dependent variable** |  |
| Y | Self-management behavior | 0=good, 1=not good |
|  | **Independent variable** |  |
| A1 | Gender | 1=male, 2=female |
| age |  | 1=“<60”, 2=“60-79”, 3=“≥80” |
| marriage | Marriage (reference group: married) | 1=unmarried, 2=married/remarried, 3=divorced/widowed |
| education | (reference group: primary school education and below) | 1=primary school and below, 2=middle school, 3=junior college and above |
| A6 | Occupation (reference group: retired) | 1=retired, 2=employed, 3=jobless |
| Income | (reference group: ≤1000 yuan per month per people) | 1=“≤1000”，2=“1001-2000”， 3=“≥2001” |
| Insurance | Method of paying medical cost (reference group: public expense) | 1=public expense, 2=social charity/commercial insurance, 3=private expense |
| A102 | Diabetes duration (reference group:1-4 y) | 1 = “1-4”, 2 =“5-9”, 3=“≥10” |
| A112 | Complication | 1=no, 2=yes |
| A122 | Hospitalization experience | 1=no, 2=yes |
| A13 | Treatment modality (reference group: in diet) | 1=diet, 2= diet + oral hypoglycemic drug/insulin,  3= diet + hypoglycemic drug + insulin |
| A142 | Number of times of health education (reference group: 0) | 1=“0”, 2=“1-4”, 3=“≥5” |
| T5 | Knowledge | Original value |
| T11 | Belief | Original value |
| T12 | Self-efficacy | Original value |
| T16 | Social support | Original value |
